# Unsedated or On-Demand Minimally Sedated Water-Aided Outpatient Colonoscopy in Colorectal Cancer Screening and Surveillance: A Step Forward or Backward? Experience from Daily Practice in a Regional (Nonacademic) Hospital

**DOI:** 10.3390/diagnostics14222596

**Published:** 2024-11-19

**Authors:** Stijn Arnaert, Diederik Persyn, Mike Cool, Guy Lambrecht, Guido Deboever

**Affiliations:** Department of Gastroenterology, AZ Damiaan, 8400 Oostende, Belgium; stijn.arnaert@student.kuleuven.be (S.A.); diederik.persyn@azoostende.be (D.P.); mike.cool@azoostende.be (M.C.)

**Keywords:** water-aided colonoscopy, colorectal cancer screening, dynamic position change, water immersion

## Abstract

**Background:** By using optimal insertion techniques with water infusion and dynamic position changes, pain during colonoscopy is greatly reduced and the procedures can usually be performed without sedation. We investigated whether the excellent results with water-aided colonoscopy reported by experts are reproducible in daily practice in a regional hospital. **Methods:** During the year 2023, 500 consecutive outpatients 50–75 years old presenting for colorectal cancer screening and surveillance could choose between unsedated or on-demand minimally sedated colonoscopy, moderate sedation with midazolam, or deep sedation with propofol. A total of 57% opted for unsedated colonoscopy, and of those patients, 250 consecutive patients were included. The primary outcome was the feasibility of the procedure. Cecal intubation rate (CIR), pain scores, use of midazolam, and willingness to repeat future procedures in the same way were registered periprocedural. Additional outcomes were cecal intubation time (CIT), detection rate of lesions, polyp resection rate, rate of adequate bowel preparation, and volume of water aspirated during insertion. **Results:** 250 consecutive sedation-free or on-demand minimally sedated water-based colonoscopies were analyzed. The CIR was 98%. A total of 96.5% completed without sedation and 5% of the procedures were perceived as moderately painful, but none had severe pain. The willingness to repeat was 97%. The mean CIT was 8.2 min. **Conclusions:** Using water-aided insertion techniques, comfortable sedation-free, or on-demand minimally sedated colonoscopy in daily practice in a regional hospital is feasible in the vast majority of patients presenting for colorectal cancer screening and surveillance, and the willingness to repeat is very high.

## 1. Introduction

Colonoscopic removal of precancerous polyps remains the gold standard in colorectal cancer prevention. The level of participation in prevention campaigns remains below expectations in many countries worldwide [1]. A high compliance with colonoscopy recommendations in colorectal cancer screening and surveillance requires high satisfaction with the procedure. To improve patient satisfaction with colonoscopy, sedation and analgesics are frequently administrated.

Sedation practices vary from country to country, center to center, and even among endoscopists in the same center [2,3,4]. Although colonoscopies for colorectal cancer screening are considered noncomplex procedures, they are increasingly performed under deep sedation and anesthesia, usually with propofol and anesthesia-monitored care (AMC).

Dynamic position changes during insertion facilitate progress during insertion and increase the adenoma detection rate (ADR) during withdrawal by expanding luminal distention and expelling the fluid from the colonic area of interest [5,6,7,8].

Water-aided techniques use water instead of CO_2_ during insertion to distend the colon enough to visualize the way forward. Keeping the lumen minimally distended with water reduces angulations and facilitates advancement with less looping of the colonoscope. In the water immersion technique (WI), the infused water is predominantly removed during withdrawal; in the water exchange (WE) technique, the infused water and residual soil and air pockets are completely suctioned during insertion [9,10,11].

WI and especially WE outperform air or CO_2_ insufflation in reducing insertion pain [11,12,13]. In addition, with WE, a higher ADR, AADR, and sessile serrated polyp detection rate are obtained [14,15,16,17,18,19]. Probably in part due to lack of knowledge or experience with water-aided techniques, this technique is not yet routinely applied in most centers.

We investigated whether the excellent results reported by expert endoscopists with water-aided unsedated colonoscopies [10,11,12,13] are reproducible in daily practice in a regional (nonacademic) hospital. The primary outcome was the feasibility of the procedure, and for this, the cecal intubation rate (CIR), the pain scores, use of midazolam, and willingness to repeat future procedures in the same way were registered during and at the end of the procedure. Additional outcomes were the cecal intubation time (CIT), the detection rate of lesions (adenoma detection rate (ADR), advanced adenoma detection rate (AADR), and cancer), the feasibility of unsedated polypectomy, the rate of adequate bowel preparation (assessed with the Boston Bowel Preparation Scale (BBPS)), and the volume of water aspirated during the insertion.

## 2. Materials and Methods

We performed a single-center prospective observational study on the feasibility and patient satisfaction of unsedated or on-demand minimally sedated water-aided colonoscopy in outpatients presenting for colorectal cancer screening and surveillance.

### 2.1. Patient Population

**Figure 1:**
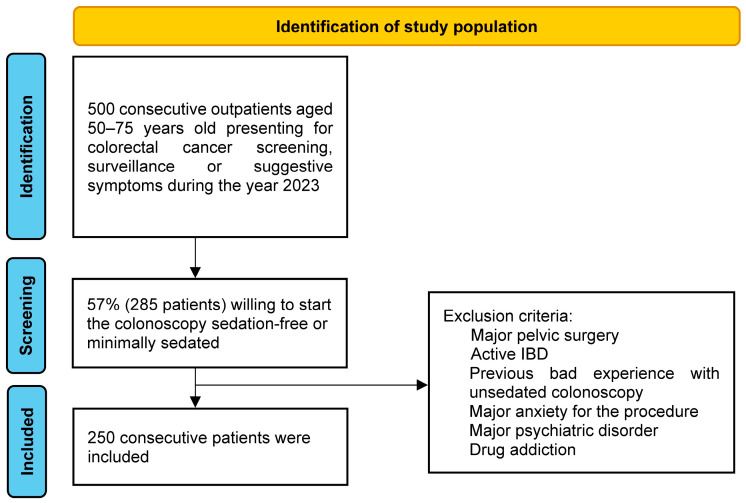
Study flow chart.

During the year 2023, 500 consecutive outpatients 50–75 years old presenting for colorectal cancer screening or surveillance (positive fecal immunological test in the national screening program, follow-up after previous polypectomy, or suggestive symptoms) could choose between unsedated or on-demand minimally sedated water-aided colonoscopy, moderate sedation with midazolam, or deeper sedation with propofol and AMC. A total of 57% were willing to start sedation-free, and 250 consecutive patients of these 57% choosing for unsedated or on-demand minimally sedated water-aided colonoscopy were prospectively analyzed (Figure 1 and Table 1).

Exclusion criteria were previous major pelvic surgery (a previous hysterectomy was not an exclusion criterium), active IBD, previous bad experience with unsedated colonoscopy, major anxiety for the procedure, major psychiatric disorder with use of high-dose psychotropic drugs, and drug addiction.

### 2.2. Bowel Preparation

All patients received a split-dose regimen of PEG (Plenvu^®^, Amsterdam, The Netherlands) for bowel preparation. Bowel preparation quality was checked by a nurse on the day clinic before the procedure was started, and, if needed, additional enemas were given to clean the colon completely.

### 2.3. Colonoscopy Procedure

**Figure 2:**
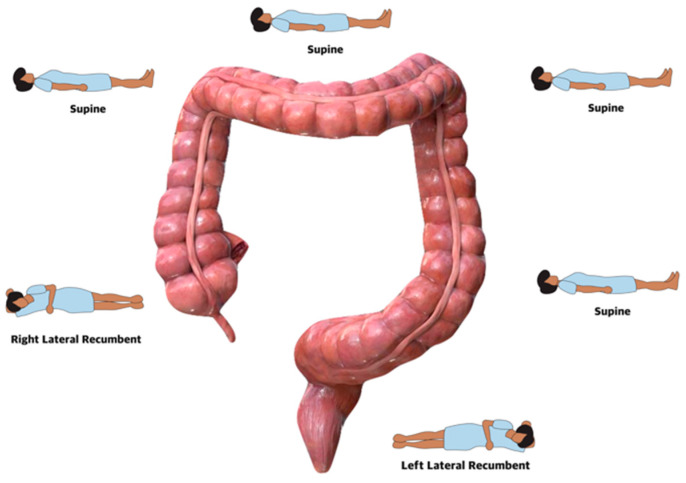
Insertion underwater.

**Figure 3:**
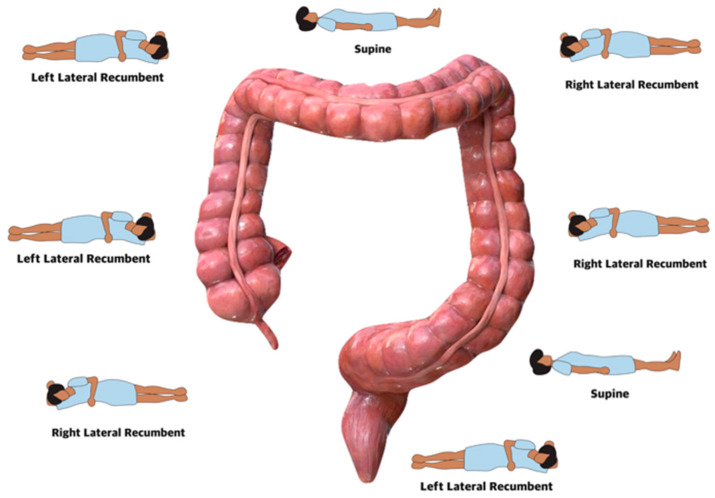
Withdrawal CO_2_.

All colonoscopies were performed by one senior endoscopist (performing about 600 colonoscopies a year) using standard HD endoscopes with variable stiffness (Olympus CF-H180I, CF-H185I, and CF-H190I, Olympus Corporation, Tokyo, Japan). Before the procedure, an intravenous catheter was placed in the day clinic. Colonoscopy was started in the left lateral position and dynamic position changes were used during insertion and withdrawal (Figure 2 and Figure 3). At the start of the procedure, butylhyoscine (10 mg) was given intravenously (IV). After aspiration of residual air and water in the rectum, starting at the rectosigmoidal junction, water (at room temperature) was injected into the colon through a water pump (Olympus O-FF 2) and progress was made underwater without insufflation. Residual soil and air pockets were aspirated while continuously injecting water with the pump, especially in the sigmoid and the left colon. On request of the patient, during the procedure, midazolam was given IV, starting with a 2 mg dose and, if needed, further titrated with 0.5 mg every 2 min. If needed to facilitate progress through angulations, external abdominal pressure was given by the nurse. After reaching the cecum, another 10 mg of butylhyoscine was given IV. During withdrawal, CO_2_ was used to distend the colonic wall.

### 2.4. Collection of Data

During the procedure, the nurse registered the CIT, the amount of water aspirated during insertion, the BBPS, and the total dose of midazolam eventually administered. Immediately after the procedure, the nurse questioned the patient on her/his willingness to repeat future procedures in the same way, and on their intraprocedural pain experience on a 4-point scale (0 = no pain at all; 1 = minimal pain; 2 = moderate pain; 3 = severe pain).

## 3. Results

### 3.1. Cecal Intubation Rate (CIR)

The CIR was 98%, slightly higher in men than in women (99% in men and 96% in women). The one male patient in whom the cecum was not reached was a very obese man weighing 125 kg presenting with a very long dolichocolon. In three female patients the cecum was not reached: one had a previous hysterectomy and a second operation with rectopexia and cystopexia, one had a very angulated and fixed diverticular sigmoid, and one had a major dolichocolon. Two of the four failed procedures were successfully completed under deep sedation with AMC.

### 3.2. Pain Scores

A total of 6% of men mentioned minimal pain and 3% moderate pain before administration of midazolam (mean pain score 1.35 on a scale of 0–3). A total of 11% of the female patients reported minimal pain and 11% moderate pain before administration of midazolam (mean pain score 1.47). If we consider only moderate and severe pain as painful, in men, 3% of the procedures were experienced as painful before the administration of midazolam, and in women 11%, but no one experienced severe pain.

### 3.3. Use of Midazolam

Midazolam was administered in 3.5% of patients: in 2% of men and in 5.5% of women. In men, a mean of 2.6 mg midazolam was administered (range 2–3 mg) and in women, a mean of 2.7 mg (range 2–3 mg).

### 3.4. Willingness to Repeat

The willingness to repeat future colonoscopies in the same way was 97%, slightly higher in men than in women (98% in men and 96% in women) (Table 2).

### 3.5. Cecal Intubation Time (CIT)

For the calculation of the CIT, the four procedures where the cecum was not reached were not taken into account. The mean CIT was 8.2 min (range 3.5–21 min): 7.4 min in men and 10 min in women.

### 3.6. ADR, AADR and Cancer

The average ADR was 64%; in the FIT+ group the ADR was 73%. Advanced adenoma was defined as an adenoma of ≥10 mm or any adenoma with high-grade dysplasia regardless of the size. The average AADR was 20%, and in the FIT+ group 30%. Five cancers were detected (three cancers in the FIT+ group) (Table 3).

### 3.7. Polyp Resection Rate

The polyp resection rate was 97% in the whole population and 95% in the population with polyps (5% of procedures with polypectomy were repeated under AMC because of large advanced sessile polyps greater than 2 cm, considered advanced procedures and removed with endoscopic mucosal piecemeal resection (EMR) or endoscopic submucosal dissection (ESD)).

### 3.8. BBPS

All patients had a BBPS > 6 (range 6–9), and the average BBPS was 8.7.

### 3.9. Aspirated Volume During Insertion

The mean aspirated volume of water during insertion was 360 mL (range 100–1150 mL).

## 4. Discussion

Water-aided unsedated colonoscopy is feasible in daily practice in a regional hospital in the vast majority of patients presenting for colorectal cancer screening and surveillance.

In our series of 250 patients, the cecal intubation rate (CIR) was 98%. The procedures could be completed without sedation in 96.5%. The procedures were, in general, perceived as comfortable, as 97% confirmed their willingness to repeat future colonoscopies in the same way without sedation or with minimal sedation on-demand. These results are in line with those reported by experts in WE in a meta-analysis: CIR 97%, on-demand sedation in 9%, willingness to repeat 91% [20].

Our results compare favorably with the results of an earlier large USA study of unsedated colonoscopy in a community-based endoscopy unit (not water-aided performed), where 85% of men and 67% of women completed the procedure sedation-free [21].

The pain-reducing effect of WE during insertion is likely due to significantly fewer loop formation compared to insertion with air or CO_2_ insufflation, as was shown in studies with magnetic endoscopic imaging [22,23]. With the patient in left lateral position, injection of water and complete removal of air allows the injected water to flow through the collapsed lumen of the sigmoid, making the sigmoid shorter and straighter. As such, there is less need for abdominal pressure and position changes during colonoscopy [24,25].

In total, 3% of men and 11% of women declared moderate pain, but none declared severe pain. In an earlier large Norwegian study on patient satisfaction with on-demand sedation for outpatient colonoscopy (not water-aided performed), the mean rate for painful colonoscopy was 34% (20% moderate painful and 14% severely painful) and the mean sedation rate was 34% [26]. In another Norwegian randomized controlled trial comparing WE versus carbon dioxide insufflation, moderate or severe pain at discharge was reported in 21% in the WE group and on-demand sedation in 6% [27].

The Performance Indicator of Colonic Intubation (PICI) combines CIR, comfort, and sedation (defined as the number of colonoscopies with cecal intubation and a high comfort score with ≤2 mg midazolam divided by the total number of all colonoscopies) [28]. Using water-aided insertion and dynamic position changes, we reached a high PICI of more than 90%, higher than the 80% level proposed by experts [28].

The mean cecal intubation time (CIT) of 8.2 min in our series compares favorably to the 12.5 min CIT in colonoscopies performed with WE in a meta-analysis [20]. The CIT was about 2.6 min shorter in men than in women (7.4 vs. 10 min), reflecting a slightly more difficult procedure in female patients. Compared to gas insufflation, water-exchange colonoscopy (WE) may require an average of 2–4 additional minutes to reach the cecum and increases the total procedure time by 2 min [18]. This 2 min additional procedure time can hardly be considered inefficient and is more than compensated by a reduced stay in hospital and discharge time [20].

The average ADR in our series was 64%. In the FIT+ population, the ADR was 73% and the AADR was 30%, similar to literature data on pooled rates of ADR and AADR in individuals with positive fecal immunological test [29,30].

The polyp resection rate was 97% in the whole population, 95% in the population with polyps, and 92% in the FIT+ population. All nonadvanced adenomas were removed during the procedure, but 3% of the procedures were repeated under AMC because of large sessile advanced adenomas of more than 2 cm, considered advanced procedures and removed with piecemeal endoscopic mucosal resection (EMR) or endoscopic submucosal dissection (ESD). We repeated these more demanding procedures in an AMC program, on the one hand, because of the difficulty of the advanced procedures, and on the other hand, because of lack of sufficient time in charged routine colonoscopy programs.

We used a WE insertion technique especially in the sigmoid and left colon, and a combination of WI and WE in the other colon segments where there were residual soil or air pockets, but without insufflation until the cecum was reached. The average volume of water aspirated during insertion was 360 mL. The intense cleansing resulted in a high average BBPS of 8.7.

Papers on predictors for difficult colonoscopy [31,32], factors to predict pain and difficulty during sedation-free colonoscopy [33,34,35,36,37,38], a score to predict the risk of high conscious sedation requirements in patients undergoing endoscopy [39], and an intubation score to predict painful unsedated colonoscopy have already been published [40]. We excluded patients with high anxiety level, previous major abdominal surgery, active IBD, previous bad experience with unsedated colonoscopy, major psychiatric disease with use of high-dose psychotropic drugs, and drug addiction. Previous hysterectomy and major obesity were not exclusion criteria in our study. However, a previous hysterectomy might be a risk factor for more difficult progress in the sigmoid colon [41,42], and the results of a recent meta-analysis confirm that obesity is a clear risk factor for difficult colonoscopy [32].

As endoscopists we have to become familiar with water-based colonoscopy. The learning curve requires at least 50 procedures [43]. At the beginning of the learning curve, not to use CO_2_ insufflation during insertion feels unusual for endoscopists who are trained to insufflate CO_2_ to distend the colon enough during insertion to visualize the way forward. The suction part is kept at the center of the lumen and the tip of the colonoscope at 11 o’clock to reduce suction-related mucosal capture. If the lumen is not visible, by injection and suction of water the direction of the fecal stream and air bubbles indicate the direction of the lumen. Educational papers and videos on water-assisted colonoscopy can be consulted online free of charge [44,45,46]. Compared to colonoscopy under AMC, sedation-free colonoscopy can sometimes require more effort for the endoscopist to pass difficult angulations, especially in the sigmoid in female patients, and at the splenic flexure. Retroflexion in the right colon is not always easy and can induce pain; in cases where retroflexion was not easy, we performed a second forward examination of the right colon.

Compared to deeply sedated colonoscopy, unsedated colonoscopy has many advantages:

- Decreased risk of sedation (hypoxemia, respiratory distress, aspiration, cardiovascular effects, and, in rare cases, death) [47,48,49,50].
- Decreased risk of injury to endoscopy workers rotating obese patients to visualize all colon segments and to approach the polyps in an ideal position.
- Efficient dynamic position changes during withdrawal increasing ADR [5,6,7,8].
- No need for personnel or anesthesiologist exclusively assigned to monitor the patient during and after the procedure
- No need for post-procedural monitoring in a recovery room
- No recovery time, reduced stay in hospital and discharge time [20]. Immediate return to full activity instead of up to 24 h after deep sedation [51,52,53].
- Avoid escort requirement.
- Reduced direct and indirect costs for the patient and health insurance (according to current reimbursement in Belgium, more than USD 200).

As such, it is a good way to achieve the goals of a safe, effective, efficient, and comfortable colonoscopy.

The present study has some limitations as it is a single-center and single-operator evaluation. The pain scores were recorded in the endoscopy suite during and at the end of the procedure but not after discharge. Some quality parameters were analyzed (CIR, ADR, AADR, pain scores, willingness to repeat), but the polyp detection rate and the sessile serrated polyp detection rate ware not registered. The number of times a retroflexed or forward second look in the right colon was performed was not registered, nor was the number of times external abdominal pressure was performed.

## 5. Conclusions

Using water-aided insertion technique and dynamic position changes, comfortable sedation-free or on-demand minimally sedated colonoscopy in daily practice in a regional hospital is feasible in the vast majority of patients presenting for colorectal cancer prevention and surveillance. The avoidance of risks of deep sedation and AMC, the reduction in cost, the reduced length of stay in the hospital, and the ability to go home without an escort favor sedation-free colonoscopy in this population. As such, sedation-free colonoscopy performed with the appropriate insertion techniques is a step forward and not backward.

## Figures and Tables

**Table 1:** Baseline characteristics.

	Male Patients	Female Patients	All Patients
Number (n)	178	72	250
Mean age (years)	63	62	63
FIT+ patients (n)	66	26	92
Other screening and surveillance patients (n)	112	46	158

**Table 2:** Colonoscopy procedural primary outcomes.

	Male Patients	Female Patients	All Patients
Caecal intubation rate (%)	99	96	98
Pain scores before administration of Midazolam (%)			
No pain Minimal pain Moderate pain Severe pain	91 6 3 0	78 11 11 0	87 8 5 0
Use of Midazolam (%)	2	5.5	3.5
Mean dosage (mg)	2.6	2.7	2.7
Willingness to repeat (%)	98	96	97

**Table 3:** Colonoscopy procedural additional outcomes.

	Male Patients	Female Patients	All Patients
Caecal intubation time (min)	7.4	10.0	8.2
Adenoma detection rate (%)			
Average ADR FIT+ patients Other screening and surveillance patients	66 73 63	58 73 50	64 73 59
Advanced adenoma detection rate (%)			
Average FIT+ patients Other screening and surveillance patients	23 31 19	13 28 5	20 30 14

## Data Availability

The data presented in this study are available on request from the corresponding author due to the unique availability of the data used in the paper.
